# The Impact of Societal Challenges on Mental Health Conditions: Implications for Research and Psychotherapy?

**DOI:** 10.32872/cpe.23554

**Published:** 2026-08-31

**Authors:** Claudi Bockting

**Affiliations:** 1 Department of Psychiatry, Amsterdam University Medical Centers, Location AMC, Amsterdam Public Health, University of Amsterdam, Amsterdam, The Netherlands; 2 Centre for Urban Mental Health, Institute for Advanced Study, University of Amsterdam, Amsterdam, The Netherlands; 3 Institute for Advanced Study, University of Amsterdam, Amsterdam, The Netherlands

**Keywords:** mental health, policy making, interventions, climate change, urban, inequality, discrimination, digital, AI

## Abstract

Mental health conditions cannot be studied in isolation and are increasingly studied as a complex system. Societal changes and challenges (the digital AI and social media era, climate change, inequality, polarization, war in Europe) have an impact on mental health, as well as the prevalence of mental health conditions. We have no idea what the accumulating effects of the combination of these societal challenges are on the prevalence of mental health conditions. At the same time, we need a mentally resilient population to deal with these societal challenges. Therefore, it is evident that mental health research as well as timely access to psychological interventions need to be prioritised. We need to study the onset and maintenance of mental health conditions, incorporating the impact of these societal challenges. Theory development is needed to identify feedback loops that can be targeted with interventions as well as policy making. The expertise of clinical psychologists is of value for policy making, and clinical psychologists might have to consider engaging more in policy making, also in dealing with societal challenges. In clinical practice, we might have to integrate the public mental health impact of current societal challenges in our diagnostics, prevention and treatment, as well as acknowledge to the individual that seeks treatment that these societal factors do affect people’s mental health.

## Highlights

- Societal changes and challenges have an impact on the prevalence of mental health conditions.
- We need a mentally resilient population to deal with these societal challenges.
- We must incorporate the impact of societal challenges on mental health in research and care.
- Clinical psychologists might have to consider engaging more in policy-making.
- In clinical practice, we might have to integrate societal challenges in our diagnostics, prevention and treatment.

Mental health conditions cannot be studied in isolation and are increasingly studied as a complex system (Borsboom, 2017; Scheffer et al., 2024). Societal challenges such as climate change are also often studied as complex systems (Scheffer et al., 2024). They are also referred to as “wicked” problems because there are no simple solutions to tackle these societal challenges. Similarly, mental health conditions can also be seen as a wicked problem, given that there are no simple solutions to manage the global mental health epidemic.

Societal changes and challenges have an impact on the mental health of people, as well as on the prevalence of mental health conditions. Among other societal challenges, we face climate change, increasing urbanisation, inequality, polarisation and war, and we live in a digital era where generative AI chatbots and social media are being massively used worldwide. At the same time, we face an ageing population in Europe and some other parts of the world. Although we have no empirical evidence for the accumulating effects of the combination of these societal challenges on mental health problems and mental health conditions as yet, there is evidence that single societal problems do affect the prevalence of mental health conditions (e.g., Brandt et al., 2026; Campbell et al., 2021; Charlson et al., 2019; Fassi et al., 2024; Gee et al., 2007; Lea et al., 2014; Lewis et al., 2015; Niazi et al., 2026; Obradovich et al., 2018; Radua et al., 2024; Seedat et al., 2009; van der Wal et al., 2021).

For climate change, for instance, an increased temperature over years was associated with a higher prevalence of mental health problems and an increase in suicidal behaviour and suicide (e.g., Obradovich et al., 2018; Radua et al., 2024). A meta-analysis and systematic review showed a link between climate change and increased prevalence of anxiety (31%), depressive disorders (28%), and posttraumatic stress disorder (23%), although heterogeneity is high (Niazi et al., 2026). Identified high-risk groups were women, low-income groups, people whose livelihood depends on climate, and Indigenous groups. Interestingly, protective factors were identified as well, such as social support and self-efficacy. These factors are potentially modifiable with psychological interventions. However, for now there is limited evidence that these interventions do work in climate-related mental health problems (Brandt et al., 2026). We need global research to examine the effect of psychological interventions, including interventions guided by non-specialists in these settings (i.e., task sharing).

In addition, our living conditions have changed and will change even further globally. We are becoming increasingly urban species, with an estimate that in less than 25 years, 68% of the world population will live in urban settings (Our World in Data, 2023). Urbanicity is associated with a higher prevalence of common mental health conditions (i.e., anxiety, depressive-, substance use disorders) in countries where at least 50% of the population is living in urban settings (van der Wal et al., 2021).

A population that is confronted with war tends to experience an increase in the prevalence of mental health conditions, also affecting the next generation (e.g., Charlson et al., 2019). Further, inequality and discrimination in society affect the prevalence of common mental health conditions. In women, depression and anxiety are twice as common as in men, and the prevalence differs per country (Campbell et al., 2021; Seedat et al., 2009).

Racial discrimination has also been linked to a higher prevalence of common mental disorders (e.g., Gee et al., 2007; Lewis et al., 2015). In LGBTQ+ people, prejudice, stigma, discrimination, as well as the consequences of discrimination (violence), are associated with poor mental health throughout life (e.g., Lea et al., 2014).

In the meantime, we live in a rapidly changing world where generative AI chatbots and social media are massively used globally. Although it is difficult to study the causal role of social media on mental health, a systematic review and meta-analysis showed that social media use is linked to more internalising mental health problems in young community populations as well as clinical populations (Fassi et al., 2024). AI most likely optimises the algorithms that personalise social media content to the individual even more. We must study how this affects mental health as well as the prevalence of mental health conditions. Globally, widely used generic chatbots such as ChatGPT, which are not designed to deal with mental health problems, are often used for emotional support (Bockting et al., 2026). Given that currently we lack independent research to examine the public mental health effects, it is difficult to estimate the effect on the prevalence of mental health conditions globally.

Especially in societal challenging time, we need a mental resilient population to deal with these societal challenges. Even more so, in an ageing Europe, we are confronted with a shrinking workforce. Young people are essential to sustaining economic growth and health care, but also to contribute to tackling some of the societal challenges. Unfortunately, the rise of mental health conditions affects young people to a large extent (e.g., Piao et al., 2022). Therefore, prioritising prevention and timely treatment of common mental health conditions is evidently necessary, including from an economic perspective. From a complex systems science perspective, all key drivers that impact mental health need to be incorporated in the system. Incorporating the impact of these societal challenges when we study the onset and maintenance of mental health conditions is a logical next step. Theory development is needed to identify feedback loops that can be targeted with interventions as well as policy making on individual-, group-, and societal level (Bockting, in press; Fried, 2026).

The expertise of clinical psychologists is also of value for policy making, and clinical psychologists might have to consider engaging more in policy-making (and vice versa), including policy that is focused on societal challenges. In clinical practice, we might have to integrate the public mental health impact of current societal challenges in our diagnostics, case conceptualisation and treatment, as well as acknowledge to the individual that seeks treatment that these societal factors do affect many people’s mental health, not only this individual. 

**Conclusion**

In this editorial, I propose that we should study mental health conditions as a “wicked" problem, just like societal challenges such as digital AI/social media, climate change, inequality and discrimination, polarization and urbanisation. These societal challenges are linked to an increase in the prevalence of mental health conditions, but we have no idea what the accumulating effects of the combination of these societal challenges are. Given that we need a mental resilient population to deal with these societal challenges, we need to prioritise mental health research as well as timely access to psychological interventions. Clinical psychologists have a lot of knowledge that can contribute to policy-making, in addition to prevention, treatment, and research. For research, we need to incorporate the impact of these societal challenges on mental health conditions in our models and research. In clinical practice, we might have to integrate the impact of societal challenges in our diagnostics and treatment.
